# Supplementary material for: Investigating Serum and Tissue Expression Identified a Cytokine/Chemokine Signature as a Highly Effective Melanoma Marker
Source: Cancers (Basel). 2020 Dec 8;12(12):3680. doi: 10.3390/cancers12123680 (PMC7762568; doi:10.3390/cancers12123680)
Supplement: Supplementary file 1 [file cancers-12-03680-s001.pdf]

cancers

MDPI

Supplementary material

Investigating Serum and Tissue Expression Identified a Cytokine/Chemokine Signature as a Highly Effective Melanoma Marker

Marco Cesati, Francesca Scatozza, Daniela D'Arcangelo, Gian Carlo Antonini-Cappellini, Stefania Rossi, Claudio Tabolacci, Maurizio Nudo, Enzo Palese, Luigi Lembo, Giovanni Di Lella, Francesco Facchiano and Antonio Facchiano

Table S1. Serum expression general data in all controls (male + female).

Cytokine	N. of values	Min	25% Percent	Median	75% Percent	Max	Average	Std. Dev.	Normal distrib.
**IL-1b**	85	0.11	0.38	0.53	0.8	13.05	0.86	1.69	No
**IL-1RA**	121	1.08	12.92	26.77	41.43	495.76	38.10	60.69	No
**IL-2**	19	0.19	2.38	3.45	10.12	31.77	7.69	8.83	No
**IL-4**	135	0.23	2.4	2.95	3.67	7.5	3.03	1.07	No
**IL-5**	30	0.34	1.7	2.77	4.69	67.9	7.54	14.73	No
**IL-6**	21	1.63	3.97	5.37	6.63	43.29	7.96	9.35	No
**IL-7**	81	0.26	1.32	2.24	3.96	126.91	4.74	14.08	No
**IL-8**	128	1	4.84	6.63	8.67	47.92	8.11	5.93	No
**IL-9**	135	2.64	34.72	45.58	55.5	102.58	46.14	18.65	Yes
**IL-10**	39	0.18	3.26	7.08	9.98	1218.15	71.50	237.54	No
**IL-12(p70)**	110	0.63	8.75	15.74	30.61	1723.01	45.14	172.48	No
**IL-13**	27	0.34	1.08	2.43	5.3	85.16	7.05	16.46	No
**IL-15**	2	1.67	26.95	52.22	77.5	102.77	52.22	71.49	No
**IL-17**	108	0.06	7.45	16.72	27.34	74.21	17.89	12.71	No
**Eotaxin**	132	7.29	65.7	95.28	134.38	471.47	108.95	67.72	No
**FGF-2**	129	4.22	24.53	32.68	39.64	107.83	33.23	14.31	No
**G-CSF**	92	0.42	3.47	4.73	6.86	82.39	5.86	8.41	No
**GM-CSF**	48	0.17	4.95	10.21	20.92	79.5	14.90	15.03	No
**IFN-γ**	136	3.52	14.21	19.1	43.12	533.04	51.19	74.66	No
**IP-10**	136	30.06	303.72	438.69	659.71	1697.24	518.32	332.07	No
**MCP-1(MCAF)**	47	1.39	9.94	18.59	26.72	172.11	22.37	25.41	No
**MIP-1a (CCL3)**	133	0.09	1.33	1.78	2.26	10.31	1.92	1.06	No
**MIP-1b (CCL4)**	136	6.29	37.17	54.36	66.32	109.96	54.74	21.68	Yes
**PDGF-BB**	135	83.69	879.78	1603.74	2117.68	5973.83	1662.24	1065.25	No
**RANTES (CCL5)**	136	581.91	7639.38	11353.34	15467.61	40521.71	12123.35	7349.35	No
**TNF-α**	120	1.79	11.94	16.4	27.16	190.28	21.75	21.46	No
**VEGF**	135	1.32	31.27	59.75	99.52	383.94	74.67	61.44	No

Table S2. General data on serum expression in all melanoma patients (male+female)

Cytokine	N. of values	Min	25% Percent.	Median	75% Percent.	Max	Average	Std. Dev.	Normal distrib.
**IL-1b**	44	0.07	0.51	0.65	0.87	10.09	1.00	1.50	No
**IL-1RA**	75	0.74	10.58	17.83	35.39	340.17	32.28	46.41	No
**IL-2**	13	0.32	1.9	2.14	4.51	10.35	3.38	2.87	No
**IL-4**	95	0.07	2.31	2.88	3.53	5.43	2.89	1.03	Yes
**IL-5**	25	0.58	1.7	2.34	3.16	10.43	2.73	2.21	No
**IL-6**	16	0.14	0.83	3.17	5.84	31.83	5.49	8.03	No
**IL-7**	48	0.35	1.32	2.24	3.85	16.09	3.08	2.93	No
**IL-8**	83	2.02	5.05	6.4	9.83	27.81	8.08	4.79	No
**IL-9**	93	1.86	29.76	42.05	55.61	234.35	45.98	27.47	No
**IL-10**	22	0.34	2.33	4.56	6.93	16.26	5.11	3.71	No
**IL-12(p70)**	62	1.96	8.41	16.07	34.03	63.5	21.80	16.29	No
**IL-13**	19	0.06	1.47	2.99	4.16	13.98	3.49	3.36	No
**IL-15**	4	8.12	15.46	30.11	45.14	53.64	30.50	21.09	-
**IL-17**	73	0.31	7.29	13.1	22.7	45.8	15.95	11.16	No
**Eotaxin**	95	1.3	70.84	106.28	153.95	378.78	116.35	73.18	No
**FGF-2**	88	1.73	23.08	30.01	38.18	162.23	31.89	18.65	No
**G-CSF**	40	0.79	3.45	5.41	7.37	16.64	5.93	3.52	No
**GM-CSF**	46	0.54	7.15	10.63	16.21	121.29	14.99	18.08	No
**IFN-γ**	86	7.45	15.68	23.2	41.53	241.76	45.05	52.79	No
**IP-10**	94	55.76	341.37	501.41	768.25	9755	783.51	1147.09	No
**MCP-1(MCAF)**	30	2.1	5.24	12.49	26.72	123.1	18.58	22.35	No
**MIP-1a (CCL3)**	95	0.09	1.34	1.74	2.29	16.42	1.92	1.67	No
**MIP-1b (CCL4)**	94	4.69	34.22	56.26	76.59	385.12	61.41	44.75	No
**PDGF-BB**	94	33.55	713.58	1033.41	1742.26	5470.53	1325.42	957.25	No
**RANTES (CCL5)**	94	179.27	5143.95	8735.27	13839.75	29388.47	9751.18	6845.14	No
**TNF-α**	65	2.98	13.15	18.06	27.44	76.09	22.11	14.20	No
**VEGF**	92	2	32.82	57.98	114.34	330.16	76.52	62.07	No

Table S3. Correlation of the serum expression with Breslow thickness in male melanoma and in female melanoma.

Cytokines	Melanoma female			Melanoma male		
	N. of pairs	Spearman R	P value (2-tailed)	N. of pairs	Spearman R	P value (2-tailed)
**IL-1b**	20	0.07	0.78	22	0.05	0.83
**IL-1Ra**	36	0.09	0.62	35	-0.16	0.37
**IL-2**	8	-0.55	ns	4	0.95	ns
**IL-4**	44	0.24	0.12	45	-0.24	0.12
**IL-5**	17	0.02	0.95	7	-0.17	ns
**IL-6**	10	0.12	ns	5	0.36	ns
**IL-7**	25	0.23	0.28	21	0.40	0.07
**IL-8**	36	-0.23	0.17	41	-0.17	0.28
**IL-9**	43	0.18	0.24	44	-0.34	**0.02**
**IL-10**	12	-0.33	0.29	9	0.05	ns
**IL-12(p70)**	27	0.04	0.83	33	-0.06	0.75
**IL-13**	11	0.20	0.55	8	-0.48	ns
**IL-15**	3	1.00	n.s	0	0	n.s
**IL-17**	36	0.25	0.14	32	-0.15	0.41
**Eotaxin**	44	0.14	0.36	45	-0.03	0.84
**FGF-2**	41	0.21	0.18	42	-0.12	0.46
**G-CSF**	17	-0.01	0.96	20	0.14	0.57
**GM-CSF**	24	-0.47	**0.02**	18	-0.32	0.19
**IFN-γ**	40	0.17	0.28	42	-0.09	0.59
**IP-10 (CXCL10)**	44	0.22	0.15	44	-0.14	0.38
**MCP-1(MCAF)**	15	0.30	0.27	14	0.40	0.16
**MIP-1a (CCL3)**	44	0.16	0.30	45	-0.29	**0.05**
**MIP-1b (CCL4)**	44	0.22	0.16	44	-0.27	0.07
**PDGF-BB**	44	0.08	0.59	44	-0.28	0.07
**RANTES (CCL5)**	44	-0.21	0.17	44	-0.16	0.29
**TNF-α**	29	0.39	**0.04**	33	0.18	0.32
**VEGF**	43	0.01	0.97	43	-0.03	0.83

Table S4. Correlation of the serum expression with age, in female controls compared to female melanoma.

	Female controls			Female melanoma		
**Cytokines**	**N. of Pairs**	**Spearman R**	**P value (2 tails)**	**N. of Pairs**	**Spearman R**	**P value (2 tails)**
**IL-1b**	32	0.43	**0.02**	22	0.05	0.82
**IL-1Ra**	50	0.25	0.07	38	-0.04	0.80
**IL-2**	7	-0.11	ns	9	0.15	ns
**IL-4**	53	0.00	0.99	47	-0.13	0.38
**IL-5**	11	0.22	0.50	18	-0.04	0.88
**IL-6**	9	-0.01	ns	11	0.06	0.88
**IL-7**	29	0.72	**< 0.0001**	27	-0.04	0.82
**IL-8**	51	-0.03	0.83	39	-0.37	**0.02**
**IL-9**	53	-0.04	0.78	46	0.00	1.00
**IL-10**	14	0.41	0.15	13	0.02	0.95
**IL-12(p70)**	45	0.39	**0.01**	29	0.02	0.93
**IL-13**	5	0.10	ns	11	0.31	0.34
**IL-15**	-	-	-	4	-0.95	ns
**IL-17**	44	0.25	0.10	38	-0.08	0.61
**Eotaxin**	51	0.04	0.77	47	-0.12	0.43
**FGF-2**	51	-0.15	0.29	43	0.01	0.93
**G-CSF**	35	-0.18	0.29	19	-0.32	0.18
**GM-CSF**	19	-0.43	0.06	26	-0.07	0.74
**IFN-γ**	53	0.26	0.06	42	-0.12	0.43
**IP-10 (CXCL10)**	53	0.02	0.91	47	0.17	0.25
**MCP-1(MCAF)**	18	0.20	0.42	16	0.44	0.09
**MIP-1a (CCL3)**	52	0.23	0.10	47	-0.28	0.06
**MIP-1b (CCL4)**	53	0.15	0.29	47	-0.10	0.51
**PDGF-BB**	53	-0.15	0.29	47	-0.28	**0.05**
**RANTES (CCL5) ((CCL5)(CCL5)**	53	-0.13	0.34	47	-0.23	0.11
**TNF-α**	46	0.34	**0.02**	31	-0.14	0.47
**VEGF**	53	0.30	**0.03**	46	0.08	0.58

Table S5. Correlation of the serum expression with age, in male controls compared to male melanoma.

Cytokines	Male controls			Male melanoma		
	N. of Pairs	Spearman R	P value	N. of Pairs	Spearman R	P value (2-tailed)
**IL-1b**	40	0.08	0.62	22	-0.11	0.63
**IL-1Ra**	58	0.31	**0.02**	37	-0.09	0.59
**IL-2**	8	0.02	ns	4	0.32	ns
**IL-4**	64	0.26	**0.04**	48	0.01	0.96
**IL-5**	12	0.58	**0.05**	7	-0.58	ns
**IL-6**	8	0.61	ns	5	0.05	ns
**IL-7**	41	0.15	0.35	21	-0.22	0.35
**IL-8**	60	0.08	0.55	44	-0.07	0.65
**IL-9**	64	0.24	0.06	47	0.08	0.60
**IL-10**	19	0.12	0.62	9	-0.13	ns
**IL-12(p70)**	50	0.36	**0.01**	33	-0.15	0.40
**IL-13**	15	0.40	0.14	8	0	ns
**IL-15**	0	0	0	0	0	ns
**IL-17**	51	0.08	0.57	35	0.04	0.84
**Eotaxin**	63	0.23	0.06	48	0.02	0.87
**FGF-2**	60	0.12	0.35	45	-0.03	0.85
**G-CSF**	43	-0.08	0.62	21	-0.36	0.10
**GM-CSF**	23	0.31	0.15	20	-0.01	0.97
**IFN-γ**	64	0.16	0.22	44	-0.10	0.53
**IP-10 (CXCL10)**	64	0.34	**0.01**	47	0.20	0.17
**MCP-1(MCAF)**	23	0.14	0.53	14	-0.59	**0.03**
**MIP-1a (CCL3)**	63	0.18	0.16	48	-0.09	0.53
**MIP-1b (CCL4)**	64	0.45	**0.0002**	47	-0.22	0.13
**PDGF-BB**	64	0.15	0.25	47	0.03	0.84
**RANTES (CCL5) UCCL5)(CCL5)**	64	0.08	0.53	47	0.10	0.49
**TNF-α**	56	0.11	0.41	34	-0.01	0.96
**VEGF**	64	0.28	**0.02**	46	0.07	0.65

Table S6. Anova analysis of tissue expression data.

Cytokines	Normality assesment			Homogen. variances	Anova P value	Post hoc tests		
	Ctrls	Primary	Metast.			Ctrls vs. Primary	Ctrls vs. Metast.	Primary vs. Metast.
IL-1b	N	N	N	N	0.02	0.15	<0.0001	0.71
IL-1Ra	N	Y	N	N	<0.0001	<0.0001	<0.0001	1.31
IL-2	N	N	N	Y	0.61	-	-	-
IL-4	N	N	N	Y	0.14	-	-	-
IL-5	N	Y	N	Y	0.74	-	-	-
IL-6	N	Y	Y	N	<0.0001	0.06	<0.0001	0.06
IL-7	N	Y	N	N	<0.0001	<0.0001	<0.0001	0.01
IL-8	N	N	N	N	0.92	-	-	-
IL-9	N	Y	N	Y	0.33	-	-	-
IL-10	Y	Y	N	Y	<0.0001	0.0004	<0.0001	0.43
IL-12(p70)	N	N	N	N	<0.0001	<0.0001	<0.0001	0.12
IL-13	N	N	N	N	0.56	-	-	-
IL-15	N	Y	Y	N	<0.0001	0.001	1.00	0.01
IL-17	N	N	N	N	<0.0001	0.08	<0.0001	0.05
Eotaxin	N	Y	N	Y	<0.0001	0.18	<0.0001	0.06
FGF-2	N	Y	Y	N	0.005	<0.0001	0.53	<0.0001
G-CSF	N	N	N	N	<0.0001	0.001	0.30	0.006
GM-CSF	N	Y	N	Y	0.61	-	-	-
IFN-γ	N	N	N	N	<0.0001	0.03	<0.0001	0.01
IP-10 (CXCL10)	N	N	N	N	<0.0001	0.36	<0.0001	0.01
MCP-1(MCAF)	N	Y	N	Y	0.0005	0.63	0.003	0.003
MIP-1a (CCL3)	N	Y	N	Y	<0.0001	<0.0001	<0.0001	0.07
MIP-1b (CCL4)	N	Y	N	N	<0.0001	<0.0001	<0.0001	0.12
PDGF-BB	N	N	N	N	0.007	0.60	0.001	0.003
RANTES (CCL5)	N	Y	N	N	<0.0001	<0.0001	<0.0001	1.00
TNF-α	N	Y	N	N	<0.0001	<0.0001	<0.0001	0.003
VEGF	N	N	N	N	0.08	-	-	-

Table S7. Results of the SVM method applied to the serum expression dataset after age-matching. Results are similar to the analysis perfomed on unmatched data (see Table 7).

Missing values	Num. melanoma	Num. controls	Training set size	Testing set size	Predictors: Sex or Age	AUC (ROC)	Accuracy	No Info Rate	*P*- value
**Removed***	54	63	83	34	**Sex, Age**	*0.635*	*0.529*	0.53	0.80
					**Sex**	0.542	0.529	0.53	0.57
					**Age**	0.538	0.588	0.53	0.30
					**None**	*0.528*	*0.588*	0.53	0.94
**Set to 0 ****	65	65	92	38	**Sex, Age**	0.609	0.632	0.50	0.07
					**Sex**	0.706	0.605	0.50	0.13
					**Age**	0.543	0.50	0.50	0.56
					**None**	0.579	0.632	0.50	0.07

*when the missing values were removed, IL4, IL8, IL9, Eotaxin, FGF2, IFN- , IP10, MIP-1a, MIP-1b, PDGF-BB, RANTES, and VEGF were simultaneously analyzed by the SVM.

** when the missing values were set to 0, all 27 cytokines/chemokines were simultaneously analyzed by SVM.
